# Autonomous self-healing structural composites with bio-inspired design

**DOI:** 10.1038/srep25059

**Published:** 2016-05-05

**Authors:** Eleonora D’Elia, Salvador Eslava, Miriam Miranda, Theoni K. Georgiou, Eduardo Saiz

**Affiliations:** 1 Centre for Advanced Structural Ceramics (CASC), Department of Materials, Imperial College London, UK; 2 Department of Chemical Engineering, University of Bath, Bath, UK; 3 Department of Materials, Imperial College London, UK

## Abstract

Strong and tough natural composites such as bone, silk or nacre are often built from stiff blocks bound together using thin interfacial soft layers that can also provide sacrificial bonds for self-repair. Here we show that it is possible exploit this design in order to create self-healing structural composites by using thin supramolecular polymer interfaces between ceramic blocks. We have built model brick-and-mortar structures with ceramic contents above 95 vol% that exhibit strengths of the order of MPa (three orders of magnitude higher than the interfacial polymer) and fracture energies that are two orders of magnitude higher than those of the glass bricks. More importantly, these properties can be fully recovered after fracture without using external stimuli or delivering healing agents. This approach demonstrates a very promising route towards the design of strong, ideal self-healing materials able to self-repair repeatedly without degradation or external stimuli.

Natural and synthetic materials often degrade during use or are subject to unexpected requirements beyond their capabilities. The result is damage and eventually failure. Several techniques have been developed for the repair of synthetic materials and many are still under investigation from new welding or joining technologies^1^ to biomimetic adhesives^2^. These techniques require that the material is removed from service and undergoes a process that could be long and complex. One of the critical issues is to ensure that the repaired structure maintains its original capabilities. A very appealing alternative would be to develop materials able to heal damage autonomously as it appears. Even the ability to repair small damage that could grow into catastrophic failure will significantly improve performance. This is exactly what natural materials do. Nature’s structural materials from bone to shells or silk, exhibit complex, intricate architectures that have long fascinated artists, scientists, and engineers. These composite structures have evolved to provide a careful balance between strength and damage tolerance combined, in some cases, with the ability to self-repair that has proven extremely difficult to recreate synthetically^3^,^4^. In recent years, significant advances have been achieved, both in the development of strong and tough, bio-inspired hybrids^5^,^6^,^7^ and in the synthesis of self-healing materials, in particular polymers^8^ and polymer-based composites^9^. However, the integration of both fields is still a pending and potentially very rewarding challenge.

A fundamental issue hindering the development of autonomous, self-healing structural materials is the fact that “sacrificial” bonds, able to break and re-form repeatedly, are weak and do not provide load-bearing capabilities. This is the case of supramolecular polymer networks able to heal due to the presence of reversible hydrogen bonds^10^. The alternative is designing materials with stronger bonding that, however, require external stimuli such as light or heat in order to catalyze self-repair (induced healing), or composites in which healing agents are encapsulated in the structure and liberated upon fracture (autonomous healing)^11^. In the latter case, the encapsulation can act as reinforcement and these materials can reach high strengths but are not able to heal repeatedly as the reinforcement breaks and the healing agent is “used”. An alternative is the use of microvascular networks that enhance the delivery of healing agents but require continuous availability of healants to allow multiple healings and therefore work better in coatings^12^. In general, it could be said that the mechanical performance of self-healing materials remains below that of “standard” engineering composites. The problem remains on how to create a strong and tough self-healing material able to self-repair multiple times without using external stimuli or compromising performance. At this point it is also important to note that when reviewing the literature healing can mean different things from the “refill’ of cracks growing in the structure to the ability to rejoin cut surfaces and recover strength. Different healing behaviors can be required in different situations (for example the ability of refilling small damage “in service” before it grows into catastrophic failure could be useful in many structural applications) and it is possible that in the future healing abilities will have to be tailored to the function in the same way that happens with the mechanical properties (strength, toughness, fatigue resistance).

Natural examples indicate that it is possible to combine strength and fracture resistance with the ability of self-repair. Furthermore, they suggest that healing capabilities can be incorporated at different length scales into complex structures as a way to increase fracture resistance. For example, Nature often uses stiff/soft brick-and-mortar composites where the soft, self-healing component often acts as a thin mortar layer that can control the sliding of the hard building blocks, rebuild the interface and maximize toughness. A good example is bone where, at the tissue level, mineralized fibrils are arranged in a multilayered stacked structure bonded with interfibrillar non-collagenous proteins^13^. Upon fracture, these proteins are able to dissipate energy contributing to the fracture resistance and imparting self-healing properties through their sacrificial bonds that can reform after fracture^14^.

Recent experiments have demonstrated the formation of reversible bonds between organic and inorganic phases at the molecular and nanometer-scale in synthetic systems^15^,^16^. However, in order to fabricate materials in practical dimensions replicating nature’s approach, it will be necessary to create interfaces able to heal at micro to macroscopic scales. The challenge could seem deceptively simple as there are reversible adhesives available commercially; but these adhesives have severe shortcomings for this application. One is passivation after exposure to the environment; the other is the fact that they cannot heal microscopic interfacial defects created during fracture. As a result, healing is always incomplete or requires an external activation such as with heat and pressure. To use this concept in the design of a ceramic-based composite able to heal fully and autonomously it is necessary to direct crack propagation along the interfaces and to design a system in which these interfaces are able to reform seamlessly and autonomously after fracture.

In this work we propose to explore a simple design idea: to use nacre-like structures in which hard, inorganic bricks are bonded by thin, shear-thickening interfaces that will behave as a “solid” during fracture but will “flow” and reform when stress is removed. We implement this design in a simple system (glass bricks joined by thin poly(borosiloxane), PBS layers) to show how by confining the soft component in a very thin layer, it is possible to retain significant strength, increase toughness and, more importantly, achieve repeated, complete and fully autonomous healing. Our goal is to use this simple model system to define the key properties of the self-healing layer and to assess the potential of the approach.

## Background

**Figure 1:**
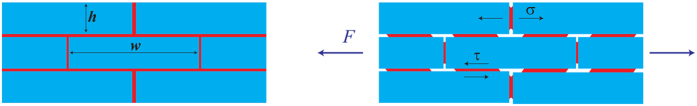
Schematic of a simple brick and mortar structure and its deformation under tension. In this case, the structure is symmetric and brick overlap corresponds to half its length.

Brick and mortar structures are present in many natural and synthetic composites. Several theoretical analyses have been published on the mechanical performance of these materials. When designing a brick and mortar structure able to self-repair it is necessary to ensure that cracks propagate through the self-healing interfaces. Irreversible brick fracture will result in decrease of the healing abilities. The use of longer bricks will promote strength but, above a critical aspect ratio (*w/h*)_*opt*_ roughly proportional to the ratio between the interfacial shear strength and the brick fracture strength, brick fracture will occur. We can use a simple model and assume that the interfacial thickness is much lower than the brick thickness and that the bricks overlap is half their length (Fig. 1). For a pure tensile test, this ratio can be estimated as^17^:


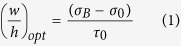


where σ_B_ is the tensile fracture strength of the bricks and σ_0_ and τ_0_ are the peak tensile and shear cohesive stresses of the mortar. For smaller aspect ratios fracture initiates and propagates through the interface and the resulting force/displacement curves are typically very similar to those recorded for natural materials^6^.

The tensile fracture strength for the optimum brick aspect ratio is 17,18:


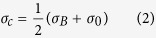


For a fixed brick aspect ratio below the optimum, increased shear strength will translate into higher composite strength. The optimum is independent on the shear strength, (Eq. (2)). This means that it is possible to reach significant strengths by combining strong bricks with relatively “soft” mortars confined in very thin interfacial layers. The composite strength can then be increased by increasing the strength of the brick. This mimics what happens in nacre, where the soft protein layers between calcium carbonate platelets act as a thin lubricant film that controls platelet sliding while retaining the materials strength^19^.

If failure is purely interfacial and not catastrophic then is it possible to also estimate the optimum work of fracture for pure interfacial failure as^17^:


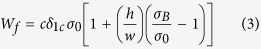


where δ_1c_ is the critical displacement at which the mortar tensile strength is maximum (σ_0_) and c is a constant that depends on the cohesive law for the adhesive interface^17^. These equations provide some simple guidelines to maximize the mechanical properties of a self-healing brick and mortar composite providing that the properties of the brick and mortar and the cohesive behavior of the interface are known.

## Experimental methods

### Polymer Synthesis

In order to synthesize the polymer, one part of dichlorodimethylsilane (DCDMS)(Sigma-Aldrich) was dissolved in two parts of diethyl ether anhydrous (VWR AnalaR NORMAPUR^®^) and hydrolyzed with two parts of distilled water. The produced polydimethilsiloxane (PDMS) was washed in a saturated solution of sodium hydrogencarbonate (Sigma-Aldrich) and the residual solvent evaporated. Polyborosiloxane (PBS) was obtained by the heat-assisted reaction of PDMS with boron oxide nanoparticles (B_2_O_3_ SkySpring Nanomaterials,Inc, average size 80 nm). The final product was dissolved in ethanol with a ratio of 80 vol% PBS : 20 vol% Ethanol.

### Thin film fabrication

For the double cantilever beam test (DCB) “sandwich” glass/polymer samples of different thicknesses (as measured using optical microscopy) were prepared using three different methods:

a) Drop casting 200 μl of the PBS solution to prepare films with thicknesses ranging between 200 to 500 μm.

b) Spin-coating the solution at 1000 rpm for 30 s to deposit layers with thicknesses varying between 50 to 200 μm.

c) Tape casting (K101Control Coater) 50 μl of solution on the glass substrate to obtain films with thickness ranging between 1 and 20 μm.

Prior to coating the glass slides (CORNING^®^ Micro Slides, Plain, Thickness 0.96 to 1.06 mm, size 75 × 25 mm) were cleaned by sonication in acetone. Tape was used to cover an area of 1 cm length along the glass slides in order to form an initiation notch inside in the interface. To fabricate the bonds the coated slides were pressed together with a force of 50 N at 40 °C for two to four days, until a homogeneous film was obtained.

### Composite fabrication

Glass slides (CORNING^®^ Micro Slides, Plain, Thickness 0.14 to 1.06 mm) were cut in bricks of different lengths, cleaned in an acetone bath, dried and dip coated with the PBS/ethanol solution. A brick/mortar-like structure was then prepared and left under a force of 50 N at 40 °C temperature for one day. The final sample was comprised of seven to 15 layers.

### Nanoindentation

The nanoindentation tests were carried out in a Nano test (Micro Materials Ltd) machine in the load control mode using a zirconia ball on a 10 μm thick polymer film prepared on a glass substrate. The holding time between loading and unloading was five minutes.

### Double cantilever beam tests

The DCB tests were carried in a Zwick testing machine with displacement rates varying between 0.05 to 20 mm/min. Because the glass samples are transparent, crack length can be directly recorded from the top using a CCD camera (DMK 23GP031 camera, Scorpion Vision Ltd). The corresponding energy release rates and critical energy release rates were calculated using simple beam theory^20^. Each interface was tested 3 to 5 times.

### Interfacial tensile and shear tests

Both tensile and shear tests of interfaces were carried out on a Zwick / Roell^®^ test machine. Tensile specimens were prepared by coating glass slides with a rectangular area of approximately 19 mm × 13 mm with PBS. For the shear test, glass slides were overlapped for an area of approximately 22 mm × 26 mm coated with a polymer layer at the overlapping interfaces. The tests were effectuated at three different speeds: 0.1 mmmin^−1^, 0.5 mmmin^−1^ and 1 mmmin^−1^.

### Three Point bending

Three point bending tests of the brick-and-mortar composites were carried out in a Zwick testing machine using a three-point bending set up with a span of 2 cm at rates varying between 0.05 and 1 mm/min. After the test the samples are removed from the rig and left healing on the laboratory bench at room temperature.

## Results and Discussion

### Self-healing interfaces at the micro to macro scales

The first step has been to test the adhesion and healing capabilities at the micro to macro scales of model interfaces formed by joining glass plates by a thin self-healing layer. We have tested three soft interfaces: a reversible adhesive, a commercially available supramolecular ureido-pyrimidone (UPy) polymer with quadruple hydrogen bonding from Suprapolix Ltd^21^, and a supramolecular siloxane polymer (poly(borosiloxane), PBS) synthesized in the laboratory. The latter, was obtained by the reaction between poly(dimethylsiloxane) (PDMS) and boron oxide nanoparticles and exhibits a structure held together by dynamic boron-oxygen bonds (supplementary information) which provide self-healing capabilities^22^. These sacrificial bonds can be broken and easily reformed spontaneously to heal the structure and the interface. In addition, PBS also exhibits good adhesion to oxide substrates and contains flexible Si-O bonds and side groups that contribute to a shear-thickening behavior: its viscosity increases with decreasing strain rate and it flows as a highly viscous liquid at low strain rates but behaves as a solid at high strain rates.

**Figure 2:**
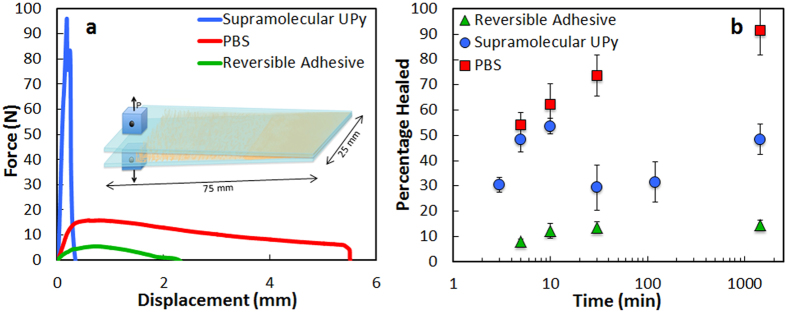
Double Cantilever Beam tests of self-healing interfaces. (**a)** Representative force/displacement curves of DCB tests (inset) carried out for 3 different interfaces at a rate of 1 mm/min (the interface thickness is much smaller than that of the substrates). For PBS and the reversible polymer, 1 mm thick glass plates have been used. The supramolecular UPy interfaces (5 μm thick) are highly adhesive and strong, resulting in the fracture of the thin (1 mm) glass substrates (measurements reported in this figure been performed with thicker-1 cm, substrates). The curve also shows a characteristic stick-slip behavior (multiple peaks) as it is commonly observed in some polymers. It could be related to inhomogneities in the coating or be an intrinsic characteristic of the polymer itself 31. The reversible adhesive interfaces (30 μm thick) exhibit very low degrees of adhesion when compared to PBS interfaces (20 μm thick). (**b)** Evolution of the degree of healing of the same sample (percentage of strength recovered) with time after closure. The reversible adhesive shows very poor recovery of the interfacial strength. The UPy polymer shows good initial healing, however, multiple healings are difficult and interfacial adhesion deteriorates after several cycles. In both cases less than 60% of the strength is recovered and it seems to saturate few minutes after closure. The shear thickening-PBS interface can instead recover full strength as the polymer flows and reforms the 20 μm interface without applying pressure.

Adhesion and healing were tested using a double cantilever beam (DCB) in which a thin polymer interface (thickness varying between 0.5 and 400 μm) was placed between two glass slides using standard techniques such as drop casting, tape casting (doctor blade) or spin coating (Fig. 2a inset)^23^. All interfaces remain stable after bonding and exhibit the same qualitative behavior during testing (Fig. 2a): after a critical stress is reached, the crack propagates and the load decreases gradually. The substrates are brought back to the initial configuration and are left healing for a set amount of time at room temperature without applying any pressure. Both, the reversible adhesive and the UPy polymer can form strong bonds but are not able to recover their full strength after bringing the plates together. In the best case, with the supramolecular UPy polymer, only up to 60% of the strength is recovered upon closure of the interface. Optical observation of the interface through the glass plates shows that during fracture microscopic defects (pores) are formed that remain after bringing the plates together (Fig. S6).

**Figure 3:**
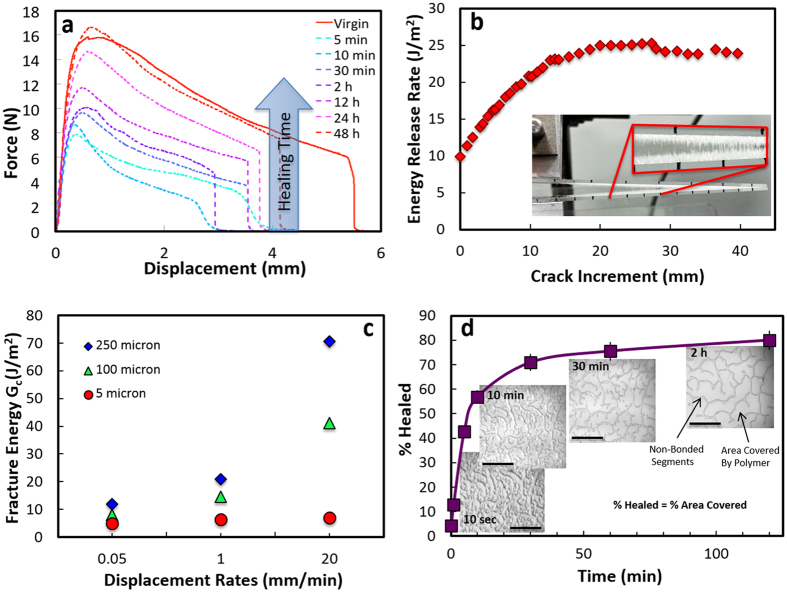
Mechanical analysis of the shear thickening interface. (**a)** Force/displacement curves from DCB tests carried out at a constant displacement rate of 1 mm/min: as the interface heals, the strength is gradually recovered. Around 50% is recovered in the first 5 minutes with complete healing in 1–2 days. (**b)** Energy release rate vs. crack length. The interface exhibits a characteristic R-curve where the capillary bridges forming behind the crack tip can reach millimeter lengths, contributing to an increase in the energy as the crack length increases up to a saturation distance of ~20 mm. (**c)** Variation of the critical fracture energy with displacement rates and thickness of the interfacial layer. Due to the shear thickening behavior of the polymer, the energy increases with displacement speed although this increase is more noticeable for thicker layers. More plastic deformation results in higher energies for thicker layers. (**d)** Percentage of healed area with time. Due to its properties the polymer flows closing macroscopic defects and reforming the interface upon closure and without external pressure. Around 80% of the interface is reformed after only 2 hours, unless otherwise specified, the interfacial thickness was 20 μm. The images are optical micrographs of the interfaces taken from the top through the glass. Scale bar in the pictures is 1 mm.

**Figure 4:**
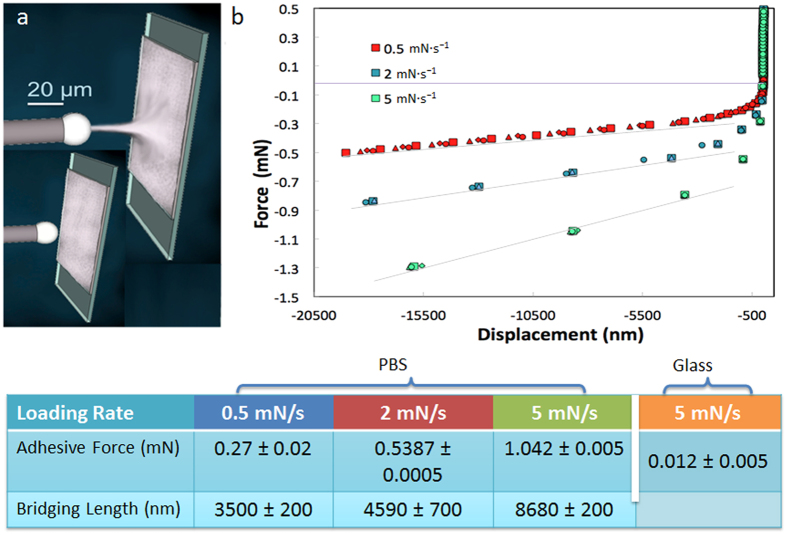
Single-filament testing. (**a)** schematic depicting the nanoindentation test. A microscopic ceramic sphere pulls away from the polymer substrate measuring the force to stretch a single polymer bridge. The bridge reaches a critical length and then breaks. (**b)** The tests (performed in load control mode) show the dependence of the force curves with the pulling rates: the higher the rate, the higher the adhesive force as a result of the shear thickening behavior of the polymer. The graph reports the results repeated on the same spot for four times (shown by the four differently shaped symbols).

In contrast, the PBS interface is able to recover its full strength after bringing the plates together. Around 50% of the strength is recovered in the first 10 minutes with complete recovery requiring times of the order of 10^3^ minutes (Figs 2b and 3a). During crack propagation in the DCB tests, small voids form ahead of the main crack front. As the crack propagates, these voids coalesce leaving behind a high density of polymer bridges linking the glass plates for distances of few millimeters behind the main crack tip (Fig. 3b). The bridges’ thickness and density depends on the thickness of the polymer layer, with thinner interfaces resulting in a higher density of finer bridges. The critical energy release rate (G_c_) also depends on the thickness of the layer and on the displacement rate (Fig. 3c) with thicker layers promoting larger energy release rates. Faster displacement rates also increase the fracture energy with the effect being more significant for thicker layers. The interface exhibits a growing resistance to crack extension with crack length (a rising resistance curve, R-curve)^24^ behavior (Fig. 3b). We have used a nanoindenter to pull a single polymer filament and probe the distance over which one of the bridges can still provide an effective capillary force. This distance can extend on the order of microns and is longer the faster the separation rate between the plates (Fig. 4).

After the DCB tests, complete healing is obtained by simply bringing the two beams back to contact even in large (up to centimeters in size) and very thin (below 5 μm) PBS interfaces without applying external stimuli or pressure. The process can be repeated multiple times. Around 80% of the interface is fully bonded in only two hours with complete healing occurring in most cases after approximately one day (Fig. 3d). Thinner interfaces heal faster than thicker ones. The healed interface remains stable with the polymer confined in a thin layer between the glass plates and can be, in some cases, stronger than the original one.

**Figure 5:**
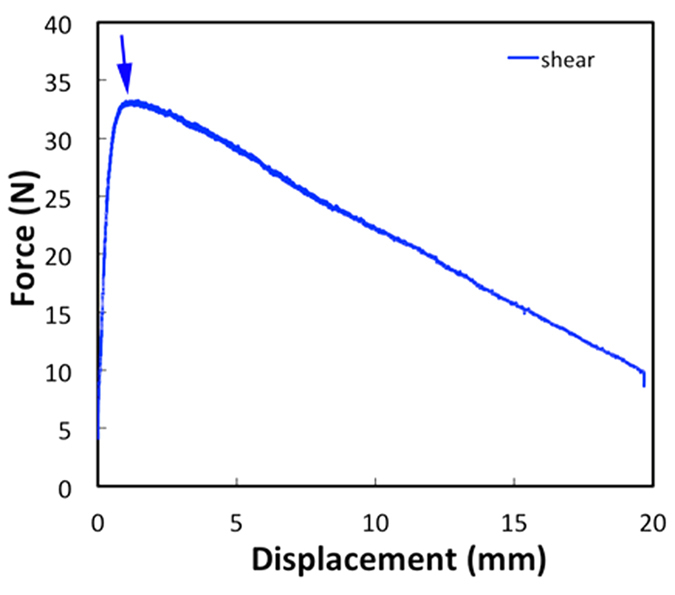
Interfacial shear test. Typical force vs displacement curve for a shear test on glass/PBS interfaces. The shear strength corresponds to the maximum in the curve. The test was carried out at constant displacement rate of 1 mm/min and the intial bonded area was 560 mm^2^.

The macroscopic tensile and shear tests of PBS interfaces also show a dependence of the corresponding strengths (maximum in the stress-strain curve) and critical displacements (displacement at the maximum stress) with the displacement rate. As expected, due to the shear thickening nature of the polymer there is a decrease of the maximum stresses for the lowest rates. In the shear tests it can be observed that once the maximum stress is reached the polymer still provides some adhesion and allows gradual sliding of the glass plates (Fig. 5). The shear strengths range between 0.07 to 0.32 MPa for displacement rates between 0.002 and 0.017 mm/s respectively.

### Self-healing brick-and-mortar structures

**Figure 6:**
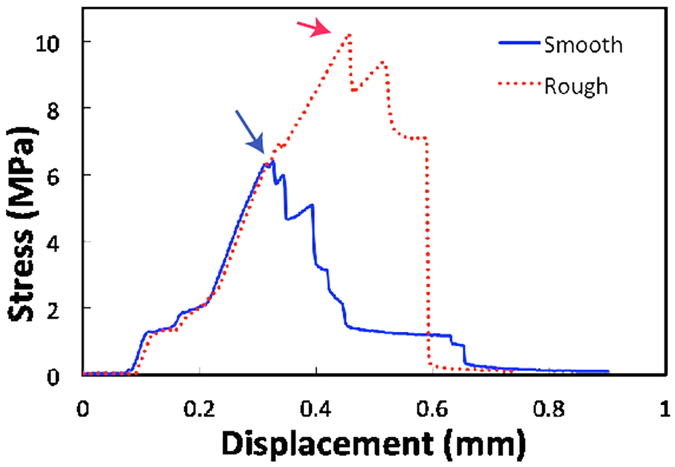
Stress vs displacement curves for thick brick and mortar composites. With brick aspect ratio h/w = 1/8 tested in three point bending for rough interfaces and smooth ones. Test carried out at 1 mm/min rate. The strength of the composite is the maximum in the strength displacement curve (arrows). The curves show the characteristic behavior that parallels that of natural materials with a large degree of inelastic deformation.

In order to demonstrate the use of the PBS self-healing interfaces in a bio-inspired composite we have fabricated model brick-and-mortar structures that mimic those of natural materials such as nacre or silk although on a much larger scale. In these engineered composites hard glass bricks with thickness, *h*, varying between 0.14–1 mm, and length, *w*, varying between 5–13 mm are held together by thin (5–10 μm thick) self-healing PBS layers. These materials were tested in bending with the load applied in the direction perpendicular to bridge alignment. Fracture initiates and propagates mostly through the interface with brick fracture being observed for bricks with aspect ratios higher than 12. The resulting force/displacement curves are very similar to those recorded for natural materials such as nacre or bone (Fig. 6)^6^. Faster loading rates result in higher strengths. Despite the “soft” nature of the polymer, the strengths can reach significant values (up to 10 MPa). Failure is not catastrophic and the resulting works of fracture (W_f_) calculated from the area under the force/displacement curves are of the order of 140 to 240 J/m^2^ with higher works of fracture for faster displacement rates.

**Figure 7:**
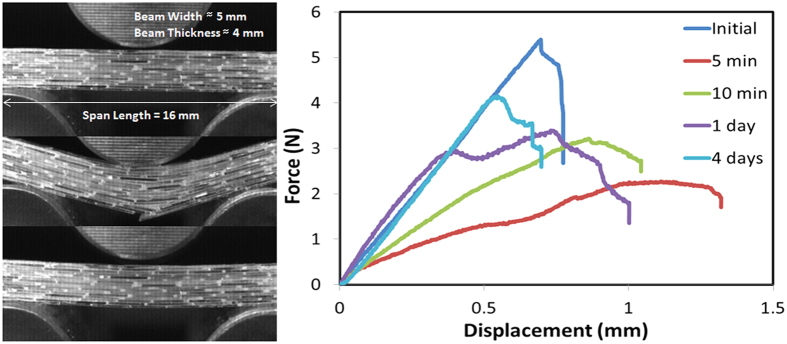
Three point bending test of thin bricks self-healing glass/polymer composites. On the left, optical images of bending before (top) during (middle) and after (bottom) fracture. The bottom picture shows how the composite autonomously reaches the initial configuration and undergoes self-healing at the interfaces The capillary bridges are able to bring the bricks together and reform the structure upon releasing the force and without applying external pressure. On the right, force vs. displacement healing curves of brick/mortar composite samples after set amount of times.

**Figure 8:**
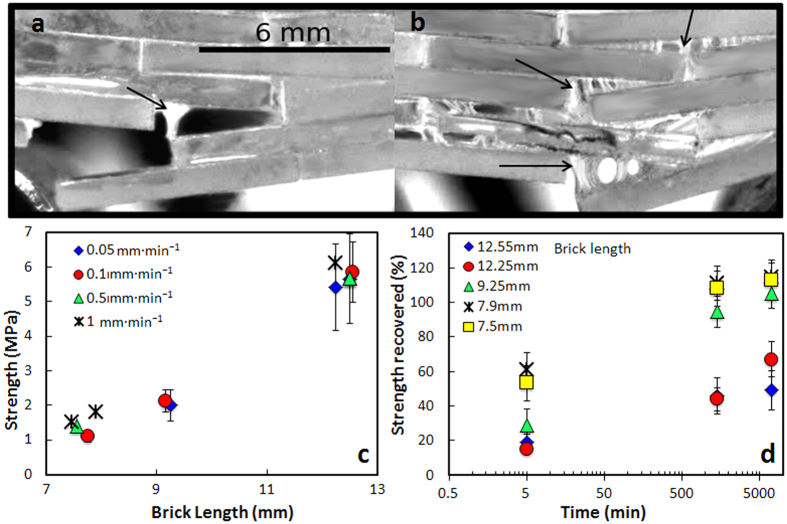
Effect of loading rate and brick length on composite healing. (**a**,**b)** *In situ* optical micrographs taken during bending tests carried out on composites formed with thicker (1 mm) bricks. Due to the shear-thickening behavior of the interface, fracture is “cleaner” during faster fracture (displacement rate of 1 mm/min) but long capillary bridges form at slow fracture (0.1 mm/min). (**c)** As a result, the fracture rate affects the strength (maximum in the stress-strain curve), although it does not influence the healing behavior which is above 90% after only 1 day. The capillary bridges bring the structure back together after removing the load without need of external pressure. (**d)** Evolution of the material’s three point bending strength with time after healing (no external pressure applied). Materials with brick aspect ratio below a critical value exhibit full interfacial fracture and recover all their strength after five days, while recovery is only partial for materials with longer bricks that undergo fracture during the first tests. As for the interfaces the materials can be stronger after healing.

As in the single interfacial tests, polymer bridges form between the hard blocks during fracture. These bridges are able to bring the structure together by themselves when the stress is removed even after very large deformations and crack propagation (Fig. 7a,b). As expected, due to the shear thickening nature of the interface, the materials tend to be stronger under faster loading rates (Fig. 8). Longer bricks also result in stronger materials (Eq. (1)). More importantly, due to the self-healing nature of the interfaces the composite strengths and works of fracture can be fully recovered after closure without applying external pressure or temperature providing that no brick fracture occurred during bending. In those cases, where brick fracture takes place, for the longer bricks, only partial recovery is achieved (Fig. 8d).

In nacre nature uses the roughness of the ceramic layers to increase strength and toughness^25^. In order to replicate nature’s strategy we have prepared composite samples with bricks roughed mechanically. The results show that a microscopically rough interface (supplementary information) leads to an increase of ~30% in strength, from 6 ± 0.5 MPa to 9.5 ± 0.5 MPa in three point bending using bricks 12.55 mm long.

## Discussion

**Figure 9:**
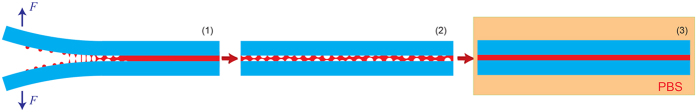
Schematic of healing and deformation process. Schematic illustrating the crack propagation and healing process for a soft self-healing interface. (1) During propagation voids nucleate in front of the crack tip, these voids generate polymer bridges linking the substrates and providing a toughening mechanism. (2) Upon closure the voids generate microscopic defects. (3) When using PBS layers, spreading of the polymer rebuilds a dense interface with properties similar to the original one.

In order to extend the brick-and-mortar designs to the fabrication of organic-inorganic composites able to heal at the macroscopic level, it is necessary to build interfaces able to heal at the micro to macroscopic scales. In this respect, the comparison of fracture and healing of three thin self-healing polymer interfaces using DCB tests has revealed a key design aspect. During crack propagation voids form and coalesce in front of the crack front as has already been observed during the failure of plastic interfaces (Fig. 9). Upon closure, some adhesives and polymers are able to reform their bonds at the molecular level but if the microscopic defects (voids) created during fracture are not completely closed, repair is not complete and full strength is not recovered. In addition, a common problem with self-healing polymers is that the surfaces become passivized after exposure to the environment following fracture due to contamination or bond reforming in the fracture surfaces. As a consequence performance will diminish with every healing cycle.

A solution to improve healing is to use an adhesive shear thickening interfacial layer able to provide effective mechanical strength during loading, whilst flowing to fully reform the interface and close microscopic defects without applying external pressure or increasing the temperature after bringing the substrates back together. To prove this concept we tested the PBS interfaces. PBS exhibits good adhesion to the glass and a characteristic viscoelastic behavior. The breaking and reforming of its chemically reversible bonds contributes to the interfacial fracture energy. As expected, thicker PBS layers promote larger energy release rates associated with an increase in the plastic energy contribution. Due to the shear-thickening nature of the interface, faster displacement rates also increase the fracture energy with the effect being more significant for thicker layers.

The nucleation and coalescence of voids during crack propagation results in the formation of thin capillary polymer bridges between the substrates. These bridges act in a similar way as the protein glue between mineralized collagen fibrils in bone, resisting the separation of the hard blocks behind the crack tip and providing a toughening mechanism that results in the development of a characteristic R-curve (Fig. 3b). The nanoindentation tests show that they provide effective bridging over distances that are of the order of micrometers (significantly higher than those recorded for natural sacrificial-bond molecules^13^,^14^) and that, unlike their natural counterpart, its extension behavior is continuous (Fig. 4).

Upon bringing the substrates back together, healing occurs thanks to an effective combination of mechanisms. First, immediate bond reformation provides some degree of recovery after bringing the glass plates together resulting in ~50% recovery in the first minutes. As for the other polymers tested, this recovery is not complete due to the remaining porosity trapped in the interface upon closure resulting from the formation of voids during crack propagation. However, in the case of PBS, this porosity is eliminated in a second step as the shear thickening polymer spreads to completely reform a defect-free interface after several hours (Figs 3d and 9). Thinner interfaces heal faster than thicker ones because the formation of a high density of thinner bridges results in smaller porosity after closure and higher surface of contact for the formation of new bonds. The healing interface can be even stronger than the original one. This increase in strength after healing has also been observed in bulk self-healing polymers^26^,^27^. It is usually attributed to the fact that in extrinsic healing systems the healing agents result to be stronger than the matrix^28^. In our case, it is possibly due to the structural re-organization of the molecules and bonds after fracture.

These results provide some insights on some of the ideal characteristics of an autonomous self-healing interface. Glues and supramolecular polymers such as UPy can provide reversible adhesion but they require pressure and passivation drastically impedes their repetitive healing. Although they are able to provide reversible bonding at the atomic-scale, they cannot heal the micro- and macroscopic defects created during fracture. Autonomous self-healing interfaces require an adhesive, shear-thickening layer able to flow and reform repeatedly without external pressure or heating. We have herein demonstrated that this can be achieved with PBS interfaces. In order to integrate a self-healing interface in the design of brick-and-mortar composites, a compromise has to be reached in order to simultaneously maximize strength, fracture resistance and healing efficiency. Using reasonable values for the brick strength (~30 MPa) and the measured σ_0_ and τ_0_ this optimum aspect ratio (eq. (1)) is of the order of ~10^2^, significantly higher than what we observed experimentally (~10). However, this calculation is based on a tensile test while our experiments are in the more complex bending configuration where brick interlocking may cause high local stresses. For lower aspect ratios fracture initiates and propagates through the interface and the resulting force/displacement curves are very similar to those recorded for natural materials^6^. As for the interfaces, and due to the shear thickening behaviour of the polymer, high loading rates result in higher strengths.

While materials with aspect ratio below the optimum value are able to fully recover their strength after one to four days (recovering more than 90% in the first 24 hours), those with higher ratio are initially stronger but can recover less due to brick fracture upon loading (Fig. 8). Laminates are the strongest design but will not recover due to layer fracture. Because in our design σ_B_≫σ_0_ the optimum composite tensile strength (eq. (2)) will be around half of the brick strength. For our composites this optimum tensile strength should be of the order of 10–30 MPa. Increasing brick surface roughness can increase friction during slides and therefore it increases the shear strength of the interface. For a fix brick aspect ratio below the optimum increased shear strength will translate into higher composite strength (the optimum is independent of the interfacial shear strength, Eq. (2)). Our maximum strength values for pure interfacial fracture in bending (~10 MPa) are in general agreement with the estimation, although slightly lower which could be due to the more complex nature of the bending tests vs a pure tensile configuration as well as the fact that our brick aspect ratios are still below the optimum. This means that despite the “soft” nature of the polymer, by using it in very thin interfacial layers, the strengths can reach significant values that can be increased by increasing the strength of the brick. This mimics what happens in nacre, where the soft protein layers between calcium carbonate platelets act as a thin lubricant film that controls platelet sliding while retaining the materials strength^19^.

Furthermore, as it has been observed in the shear tests, the PBS layers plays a “lubricant” role akin to the one of the organic phase in nacre and allows controlled interfacial sliding of the inorganic bricks promoting fracture resistance. Failure is not catastrophic and we can use eq. (3) to calculate the corresponding work of fracture (we take c ~ 1 17). The resulting maximum works of fracture W_f_ ≈ 0.07–0.35 kJ/m^2^ (considering a brick strength of the order of 30 MPa, depending on the displacement rates they increase with increasing loading rate due to the shear thickening nature of the polymer) for the optimum brick aspect ratio are reasonably close to those measured experimentally from the area under the load/displacement curve^29^, W_f_ ≈ 0.14–0.24 kJ/m^2^. These experimental values are two orders of magnitude higher than the work of fracture for glass.

More importantly, due to the self-healing nature of the interfaces these strengths and works of fracture can be fully recovered after closure without applying external pressure or temperature. The capillary polymer bridges are able to bring the structure together and heal the interface when the stress is removed even after very large deformations (Fig. 8). This result suggests that an ideal self-healing interface could be formed by a soft, shear thickening material.

**Figure 10:**
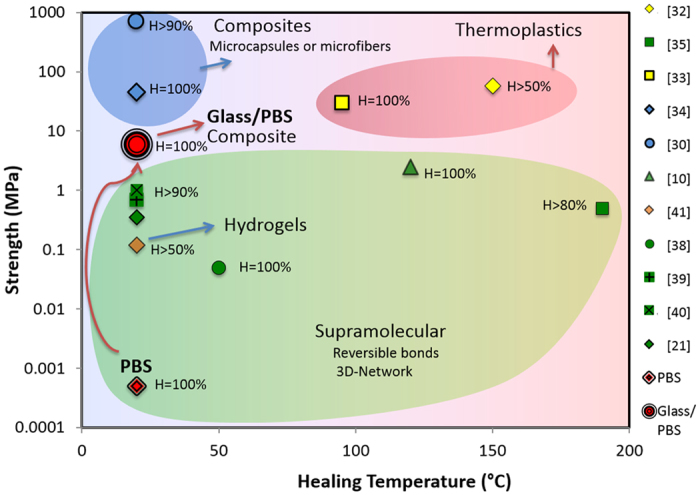
Strength vs healing temperature for self-healing materials. Composites, usually made by the incorporation in a matrix of microspheres^34^ or microfibers^30^, present good mechanical properties, and the ability to heal at room temperature. However, they do not allow multiple healing in the same spot, as the healing agent is exhausted and the reinforcement breaks. Thermoplastics instead^32^,^33^, have good mechanical properties but can heal only with external stimuli such as heat or light. Supramolecular polymers allow multiple healing due to their hydrogen bonded networks, but are generally weak^38^ or need external stimuli^10^ for the healing, making the process not autonomous^21^,^35^,^38^,^39^,^40^. Recently, self-healing hydrogels have also been tested showing strengths of the order of kPas^41^. In this work we have successfully produced a Glass/Supramolecular polymer composite (Glass/PBS) able to heal completely and autonomously at room temperature multiple times. The strength of the composite (in the order of MPa) is above three orders of magnitude higher than the weak thin supramolecular polymer interface (PBS). It approaches the strongest materials and is comparable to strong supramolecular polymers that need temperatures above 100 °C for healing.

It is useful to compare the performance of these simple model composites with current self-healing materials (Fig. 10). Thermoplastics or fiber-reinforced composites exhibit the highest strengths (from tens to hundreds of MPa) and their healing times range between hours to a few days^10^. However, thermoplastics require high temperature and fiber or capsule-reinforced composites can only repair once and not fully, since the reinforcement breaks during fracture^30^,^31^,^32^,^33^,^34^. Weaker, non-structural compounds can heal faster (tens to hundreds of minutes) but often require activation through UV light, temperature or pressure^10^,^21^,^35^,^36^,^37^,^38^,^39^,^40^,^41^. Here, we have used a biomimetic design based on self-healing interfaces built from a relatively simple polymer to create ceramic-based hybrids with strengths and recovery times comparable to those reported for structural polymer-based materials. Eventually the recovery time will be dictated by the speed at which the interface re-forms (the spreading of the interfacial polymer). Lower viscosity polymers will spread faster and provide faster healing although they may require much longer bricks to develop the optimum strengths (eqs 1 and 2). The practical implementation of this approach will eventually require the combination of modelling to select an optimized self-healing interface and brick design with existing technologies for the practical assembly of micro to macroscopic bricks from additive manufacturing to freeze casting^6^.

## Conclusions

Taking clues from natural systems we have designed self-healing composites able to repair autonomously and repeatedly, recovering all their strength at room temperature. Following nature’s approach, the “soft” and thin interfaces control the sliding of the stiff blocks and enhancing fracture resistance while promoting healing upon closure. This simple model system illustrates some key issues. For example, in a practical material, healing at microscopic level is essential and requires repair of microscopic defects created during fracture (e.g. due to the formation and coalescence of voids during crack propagation). A shear-thickening polymer able to “flow” and create capillary bridges will promote structural recovery and heal defects and reform the interface without applying external pressure even after large deformations in a way that cannot be achieved with a reversible adhesive or a stiffer supramolecular material. Nature engineers its “soft” interfaces at the chemical (bonding, rheology) and structural levels (thickness, roughness…). It also manipulates structures at multiple length scales from molecular to macro levels using very simple components. Here we have followed a similar path by using a model system with very simple components that provides useful guidelines but being still far from optimal. However, we believe that our results prove that by mimicking nature’s strategy we can open a new path towards the design of strong “ideal” self-healing composites that could use the wide palette of synthetic compounds to select optimum brick and mortar combinations.

## Additional Information

**How to cite this article**: D’Elia, E. *et al.* Autonomous self-healing structural composites with bio-inspired design. *Sci. Rep.* **6**, 25059; doi: 10.1038/srep25059 (2016).

## Supplementary Material

Supplementary Information

## References

[b1] Fernie J. A., Drew R. A. L. & Knowles K. M.. Joining of engineering ceramics. International Materials Reviews 54(5), 283–331 (2009).

[b2] Meredith H. J. & Wilker J. J.. The Interplay of Modulus, Strength, and Ductility in Adhesive Design Using Biomimetic Polymer Chemistry. Advanced Functional Materials 25(31), 5057–5065 (2015).

[b3] Bruet B. J. F. *et al.* Materials design principles of ancient fish armour. Nature Materials 7(9), 748–756 (2008). 18660814 10.1038/nmat2231

[b4] Launey M. E. *et al.* A novel biomimetic approach to the design of high-performance ceramic-metal composites. Journal of the Royal Society Interface 7(46), 741–753 (2010). 10.1098/rsif.2009.0331 PMC2874234 19828498

[b5] Studart A. R. Biological and Bioinspired Composites with Spatially Tunable Heterogeneous Architectures. Advanced Functional Materials 23(36), 4423–4436 (2013).

[b6] Munch E. *et al.* Tough, Bio-Inspired Hybrid Materials. Science 322(5907), 1516–1520 (2008). 19056979 10.1126/science.1164865

[b7] Erb R. M. *et al.* Composites Reinforced in Three Dimensions by Using Low Magnetic Fields. Science 335(6065), 199–204 (2012). 22246772 10.1126/science.1210822

[b8] Wu D. Y., Meure S. & Solomon D.. Self-healing polymeric materials: A review of recent developments. Progress in Polymer Science 33(5), 479–522 (2008).

[b9] White S. R. *et al.* Autonomic healing of polymer composites. Nature 409(6822), 794–7 (2001). 11236987 10.1038/35057232

[b10] Cordier P. *et al.* Self-healing and thermoreversible rubber from supramolecular assembly. Nature 451(7181), 977–980 (2008). 18288191 10.1038/nature06669

[b11] Mauldin T. C. & Kessler M. R.. Self-healing polymers and composites. International Materials Reviews 55(6), 317–346 (2010).

[b12] Toohey K. S. *et al.* Self-healing materials with microvascular networks. Nature Materials 6(8), 581–585 (2007). 17558429 10.1038/nmat1934

[b13] Wang R. Z. & Gupta H. S.. Deformation and Fracture Mechanisms of Bone and Nacre. Annual Review of Materials Research, 41(**41**), 41–73 (2011).

[b14] Fantner G. E. *et al.* Sacrificial bonds and hidden length dissipate energy as mineralized fibrils separate during bone fracture. Nature Materials 4(8), 612–616 (2005). 16025123 10.1038/nmat1428

[b15] Lee H., Lee B. P. & Messersmith P. B.. A reversible wet/dry adhesive inspired by mussels and geckos. Nature 448(7151), 338–U4 (2007). 17637666 10.1038/nature05968

[b16] Lee H., Scherer N. F. & Messersmith P. B.. Single-molecule mechanics of mussel adhesion. Proceedings of the National Academy of Sciences of the United States of America 103(35), 12999–13003 (2006). 16920796 10.1073/pnas.0605552103 PMC1559742

[b17] Wilbrink D. V. *et al.* Scaling of strength and ductility in bioinspired brick and mortar composites. Applied Physics Letters 97(19) (2010).

[b18] Barthelat F. Designing nacre-like materials for simultaneous stiffness, strength and toughness: Optimum materials, composition, microstructure and size. Journal of the Mechanics and Physics of Solids 73, 22–37 (2014).

[b19] Meyers M. A. *et al.* Mechanical strength of abalone nacre: Role of the soft organic layer. Journal of the mechanical behavior of biomedical materials 1(1), 76–85 (2008). 19627773 10.1016/j.jmbbm.2007.03.001

[b20] Moore D., Pavan A. & Williams J. G.. Fracture mechanics testing methods for polymers, adhesives, and composites. ESIS publication, Amsterdam; Oxford: Elsevier. xi, 375 p (2001).

[b21] van Gemert G. M. L., Peeters J. W., Söntjens S. H. M., Janssen H. M. & Bosman A. W. Self-Healing Supramolecular Polymers In Action. Macromol. Chem. Phys. 213, 234–242 (2012).

[b22] Li X. F. *et al.* Synthesis of polyborosiloxane and its reversible physical crosslinks. Rsc Advances 4(62), 32894–32901 (2014).

[b23] Krebs F. C. Fabrication and processing of polymer solar cells: A review of printing and coating techniques. Solar Energy Materials and Solar Cells 93(4), 394–412 (2009).

[b24] Anderson T. L. Fracture mechanics: fundamentals and applications. 3rd ed. Boca Raton, FL; London: CRC/Taylor & Francis. 621 (2005).

[b25] Evans A. G. *et al.* Model for the robust mechanical behavior of nacre. Journal of Materials Research 16(9), 2475–2484 (2001).

[b26] Keller M. W., White S. R. & Sottos N. R.. A self-healing poly (dimethyl siloxane) elastomer. Advanced Functional Materials 17(14), 2399–2404 (2007).

[b27] Brown E. N., Sottos N. R. & White S. R.. Fracture testing of a self-healing polymer composite. Experimental Mechanics 42(4), 372–379 (2002).

[b28] Zwaag S.v.d. Self Healing Materials: An Alternative Approach to 20 Centuries of Materials Science. Springer: Netherlands., Vol. 100, 388 (2007).

[b29] Clegg W. J. *et al.* A Simple Way to Make Tough Ceramics. Nature 347(6292), 455–457 (1990).

[b30] Pang J. W. C. & Bond I. P.. A hollow fibre reinforced polymer composite encompassing self-healing and enhanced damage visibility. Composites Science and Technology 65(11–12), 1791–1799 (2005).

[b31] Kessler M. R., Sottos N. R. & White S. R.. Self-healing structural composite materials. Composites Part A: Applied Science and Manufacturing 34(8), 743–753 (2003).

[b32] Chen X. X. *et al.* A thermally re-mendable cross-linked polymeric material. Science 295(5560), 1698–1702 (2002). 11872836 10.1126/science.1065879

[b33] Plaisted T. A. & Nemat-Nasser S.. Quantitative evaluation of fracture, healing and re-healing of a reversibly cross-linked polymer. Acta Materialia 55(17), 5684–5696 (2007).

[b34] Rong M. Z. & Zhang M. Q. Self-Healing Polymers and Polymer Composites. New Jersey: Wiley. Vol. 1, 416 (2011).

[b35] Burnworth M. *et al.* Optically healable supramolecular polymers. Nature 472(7343), 334–U230 (2011). 21512571 10.1038/nature09963

[b36] Chung C. M. *et al.* Crack healing in polymeric materials via photochemical [2 + 2] cycloaddition. Chemistry of Materials 16(21), 3982–3984 (2004).

[b37] Ghosh B. & Urban M. W.. Self-Repairing Oxetane-Substituted Chitosan Polyurethane Networks. Science 323(5920), 1458–1460 (2009). 19286550 10.1126/science.1167391

[b38] Tee B. C. K. *et al.* An electrically and mechanically self-healing composite with pressure- and flexion-sensitive properties for electronic skin applications. Nature Nanotechnology 7(12), 825–832 (2012). 10.1038/nnano.2012.192 23142944

[b39] Rekondo A. *et al.* Catalyst-free room-temperature self-healing elastomers based on aromatic disulfide metathesis. Materials Horizons 1(2), 237–240 (2014).

[b40] Chen Y. L. *et al.* Multiphase design of autonomic self-healing thermoplastic elastomers. Nature Chemistry 4(6), 467–472 (2012). 10.1038/nchem.1314 22614381

[b41] Tuncaboylu D. C. *et al.* Tough and Self-Healing Hydrogels Formed via Hydrophobic Interactions. Macromolecules 44(12), 4997–5005 (2011).

