# Supplementary material for: Autonomous self-healing structural composites with bio-inspired design
Source: Sci Rep. 2016 May 5;6:25059. doi: 10.1038/srep25059 (PMC4857079; doi:10.1038/srep25059)
Supplement: Supplementary Information [file srep25059-s1.pdf]

Supplementary Information

Autonomous self-healing structural composites with bio-inspired design

Eleonora D'Elia,[1] Salvador Eslava,[2] Miriam Miranda,[1] Theoni K. Georgiou,[3] Eduardo Saiz[1]

[1]Centre for Advanced Structural Ceramics (CASC), Department of Materials, Imperial College London, UK

[2] Department of Chemical Engineering, University of Bath, Bath, UK

[3] Department of Materials, Imperial College London, UK

Polymer characterization

Polymer structure

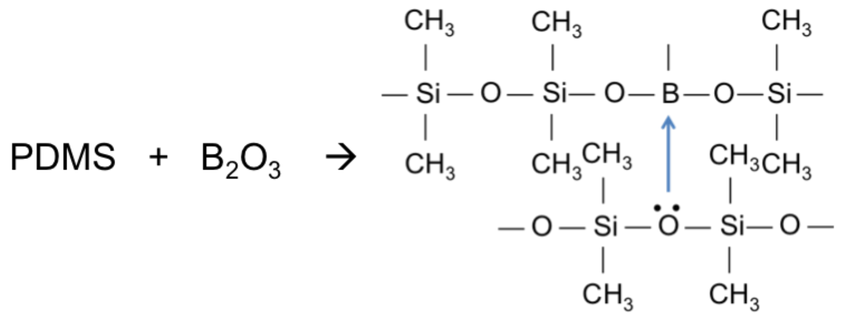

Figure S1. Supramolecular Polyborosiloxane. Cross-linking reaction between Polydimethilsiloxane (PDMS) and B_2O_3 nanoparticles results in the formation of a supramolecular aggregate with a dynamically cross-linked structure: PBS.

Table S1 Molecular weight analysis of different polymers and cross-linked specimens. The table shows the different molecular weight polymers used for the experiments and the cross-linked supramolecular self-healing samples. It is evident how the sample synthesized for the experiment starting from the hydrolysis of DCDMS show a similar molecular weight to the polymers available commercially under the name of Silly Putty®. The polydispersity index (PDI) Reported in the table shows a low polydispersity, meaning a good optimization of the process. In order to vary the molecular weight of the samples used, different polymers were purchased by Aldrich, (Particularly PDMS 25 cSt hydroxyl terminated and PDMS 750 cSt) and subsequently crosslinked with Boron Oxide nanoparticles. Polymers with higher Mw (28280 Da) resulted to be too soft for the application in this study.

	Mw (Dalton) ±100	***PDI***
25 cSt Aldrich PDMS	*755*	*1.1402*
750 cSt Aldrich PDMS	*33438*	*1.4012*
25 cSt Aldrich PBS	*1432*	*1.458*
750 cSt Aldrich PBS	*28280*	*1.332*
Synthesized PDMS	*3290*	*1.333*
Synthesized PBS	*6484*	*1.8345*
Commercially available PBS polymer	*5871*	*1.58*

Thermal Analysis

Differential scanning calorimetry (DSC) was performed on a Q100 T A Instruments calorimeter with a temperature range between -150°C and 150° at a rate of 5°C/min. At -122 it is possible to observe the presence of a peak, corresponding to the Tg of PDMS as reported in literature by Dobkowski et al. (Thermal Analysis of the Poly(Siloxane)-Poly(Tetrafluoroethylene) Coating System, 2002). The transition obtained at higher temperatures is related to the Tg of the PBS.

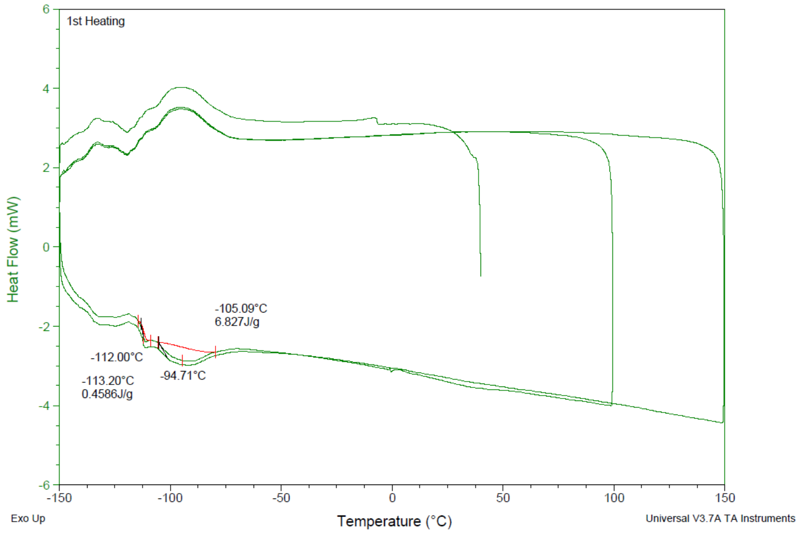

Figure S2 DSC Scan of Low Molecular weight PBS (1400 ± 100Da)

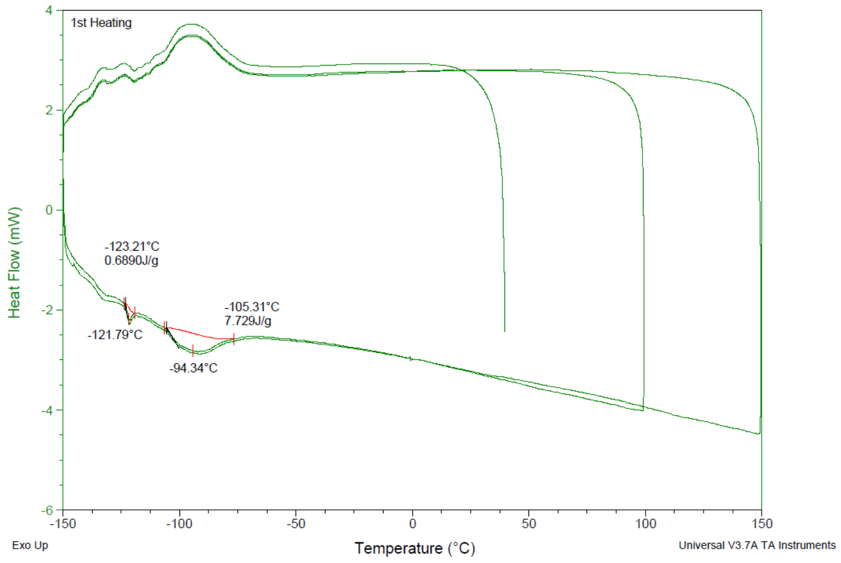

Figure S3 DSC Scan of Low Molecular weight PBS (28200 ± 1000Da)

Rheology

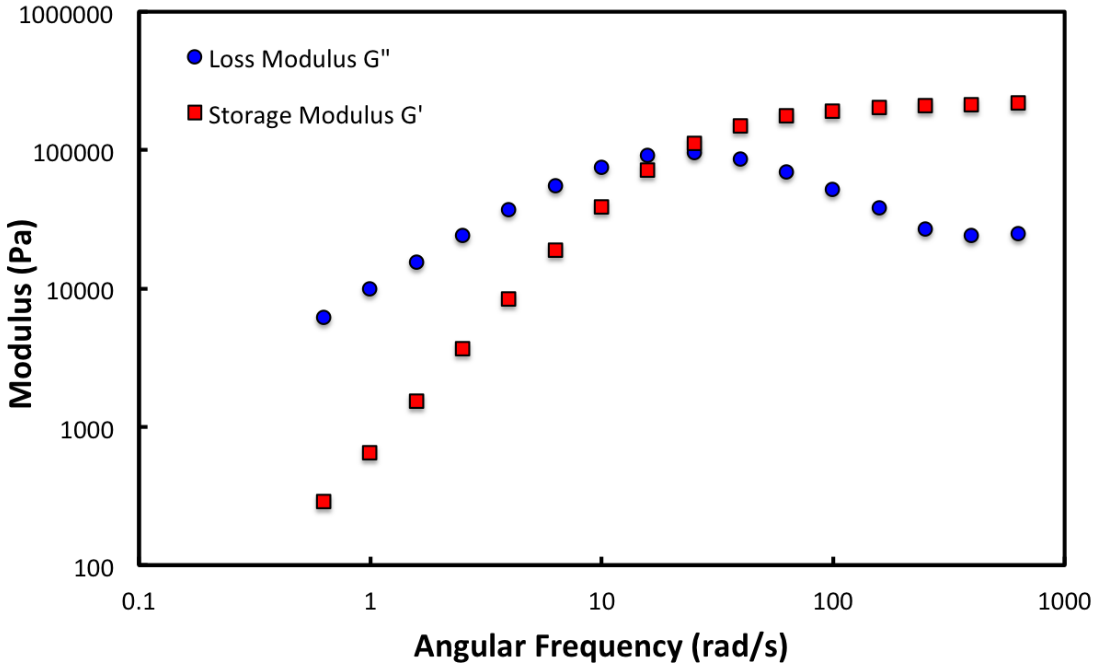

Figure S4. Rheology of the synthesized self-healing Polymer showing the shear-thickening behavior of the aggregate.

Composites

Glass brick surface

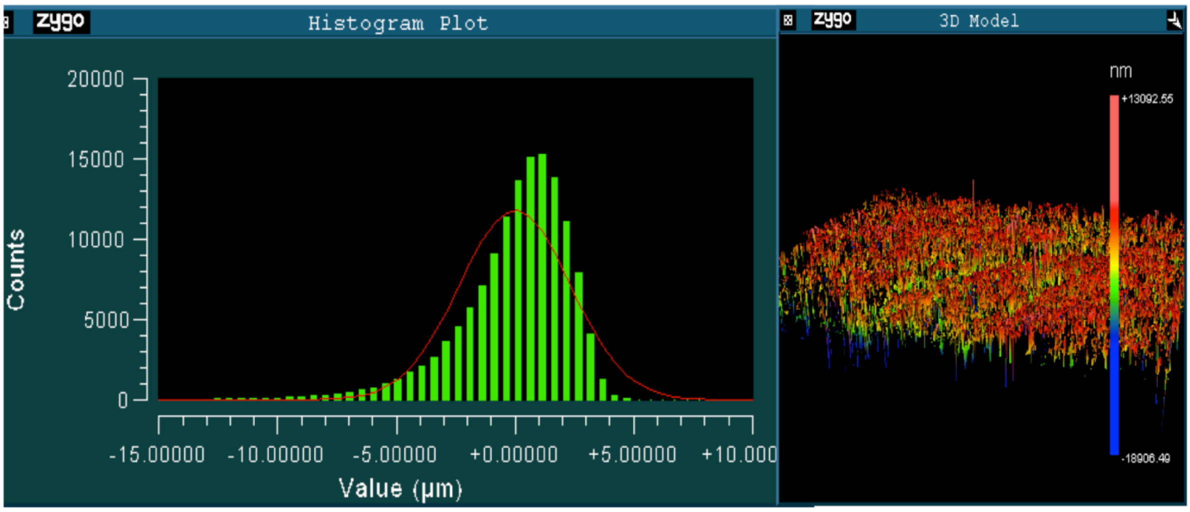

***Figure S5 Rough glass surfaces.** Brick and mortar composites have been fabricated using flat, "as received" glass surfaces as well as surfaces that have been previously roughened using sand paper. Optical interferometry results carried out on roughened glass surface showing a histogram plot and a 3d model of the asperities. The results show size of the asperities varying between approximately ±5µm.*

Interfacial porosity

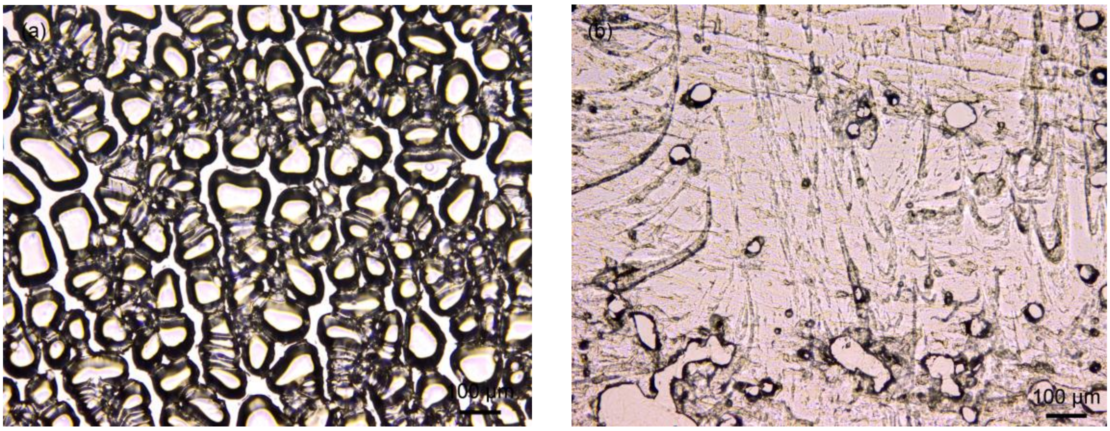

Figure S6. Residual microscopic porosity after closing a DCB test sample. Optical micrographs taken through the glass substrates showing the interfacial porosity remaining at the glass/polymer interface after bringing the glass plates together following a DCB test. The images correspond to (a) PUy and (b) reversible adhesive interfaces. A large amount of residual porosity is clearly visible in both.
